# NEWS

**DOI:** 10.7189/06.020203

**Published:** 2016-12

## Demography

➢ New figures from the US federal government shows a decline in the US birth rate and an increase in the death rate, which could lead to downwards pressure on the country’s population growth. In 2015, there were 3.98 million births in the US, a 0.3% reduction from 2014 and reversing previous gains. Despite the sharp fall in births among women aged 15–19 years, some demographers are concerned that the birth figures are lower than expected, and that the US is still to recover from the slowdown in its birthrate arising from the 2007 recession. The rise in the country’s death rate (from 729.5 deaths per 100 000 people in 2015, from 723.2 in 2014) is partly due to increases in deaths from heart disease, stroke and Alzheimer disease; and although the statistics on deaths from suicide and drug use are not yet available they are also likely to show an increase. According to Jeffrey Passel, a senior demographer at the Pew Research Center, “to keep the labour force growing, we’re going to need to have pretty healthy levels of immigration.” (*Wall Street Journal*, 15 June 2016)

➢ India is placed last on an annual retirement security index, which ranks 43 countries, including the BRICS nations, on retirement security factors including quality–of–life, health and finances. India was the poorest–scoring BRICS country in health care, mainly caused by its low expenditure on health care, high non–insured health expenses, and inadequate basic health care in rural areas. India also fared badly in the quality–of–life category, due to factors like air quality, happiness and environmental pollution. Another report from HSBC shows that 47% of working people in India have not begun to save for their retirement, or face difficulties in saving for their retirement. This is coupled with high expenses during retirement – 68% of retired people in India report borrowing or supporting other people financially, compared to 50% globally – which suggests that people aren’t saving enough for retirement. About 8.6% of India’s population is aged over 60, and the elderly population is expected to increase by 20% by 2021. (*Huffington Post India*, 20 July 2016)

➢ Almost 15% of US citizens are poor – including 20% of all children – and almost one–third of all households is headed by a single mother. These levels are higher than most other developed countries, and have proven stubbornly high even during strong economic growth. Bill Clinton enacted the last major reforms to US welfare in 1996, which capped welfare at US$ 16.5 billion a year, and put the states in charge of implementing it. Labelling it Temporary Assistance for Needy Families, he intended to make “welfare a second chance, not a way of life”, with payments conditional on the recipient looking for work, and limiting their receipt to 5 years in total. On some measures, the policy has been successful, as spending on welfare fell to US$ 11.1 billion a year (although non–cash benefits like food and housing vouchers covered any shortfalls), and fewer Americans claim welfare. However, the tough economic conditions of the past 10 years have highlighted its shortcomings for the millions still stuck in poverty. First, Mr Clinton promised guaranteed public–sector jobs to claimants, which did not materialise, and many individual states have provided inadequate training. The move toward low–grade service jobs across the US economy means that many people are stuck close to the poverty line; and even among those who fare better, the sharp withdrawal of tax credits (in effect, up to a 60% tax rate) disincentivises progress. Falling cash payments has led to a new, cash–poor, group of people who rely on vouchers rather than cash, leaving them unable to pay a phone bill or afford a haircut. Improving their outlook could mean increasing the amount of welfare paid in cash, and ensuring that states cannot plunder welfare payments. (*Economist*, 20 August 2016)

➢ According to the UK’s Overseas Development Institute (ODI), Africa’s children will comprise more than 40% of the world’s poorest people by 2030 – almost double the current amount – unless education and health care are improved. The ODI estimated that 88% of all children living on US$ 1.90/d by 2030 will be in sub–Saharan Africa – up from 50% today. The ODI calls for cash transfers, education and health care to reduce poverty, extreme inequality and alter demographic patterns, and notes the central role of education in enabling girls to gain skills, delay marriage and demand better health and reproductive care. Mr Kevin Watkins, the director of ODI, states that Nigeria – Africa’s most populous country – has the largest number of children out of school and large gender gaps in education. Unless Nigeria improves, it will distort gains within other countries in sub–Saharan Africa. Sub–Saharan Africa has experienced economic growth and made significant progress in child survival rates, and Mr Watkins calls on African leaders to invest in their young people and end child marriage (more than 10% of girls are married by age 15, and 40% are married by age 18 in sub–Saharan Africa), which prevents young women from exercising choice and realizing their potential. (*Thomson Reuters Foundation*, 24 August 2016)

➢ The current Sustainable Development Goals – which aim to end poverty, boost prosperity and promote sustainability – will end in 2030 when the world’s population will be 8.5 billion people, and just 20 years ahead in 2050, the world’s population will have increased to nearly 10 billion. For an indication of how the world could look in 35 years’ time, it is interesting to track progress from 34 years ago. In the early 1980s, the population was 4.5 billion, of whom 42% lived in extreme poverty. Today, the population has reached 7.5 billion, yet just 10% of the total population live in extreme poverty. This is largely due to China and India, who have succeeded in lifting millions of people out of poverty and improving health outcomes. This was achieved partly by strengthening institutions and promoting strong, relatively inclusive growth; and China look advantage of its “demographic dividend”, whereby the labor force grew faster than the dependent population. Today, 90% of global poverty is concentrated in countries with growing working–age populations – an important opportunity for rapid poverty reduction, provided that productive jobs can be generated. However, several middle–income countries will begin to experience a decline in the relative share of their working–age population, as higher incomes impels households to delay having children. This can be potentially be offset by the accumulated savings of an older generation producing a surge in investment, helped by improving productivity and adapting social–welfare systems. (*Project Syndicate*, 25 October 2016)

## Economy

➢ A study from Boston University and the Chinese Academy of Social Sciences found that China is a bigger presence in development finance that the major multilateral financial institutions combined, doubling the volume of global development finance over the past 10 years. In 2014, two Chinese policy banks – the China Development Bank (CDB) and the Export–Import Bank of China – have an estimated overseas loan portfolio of US$ 684 billion which is just below the US$ 700 billion from the combined western multilateral development institutions (World Bank, Asian Development Bank, Inter–American Development Bank, European Investment Bank, European Bank for Reconstruction and Development, and African Development bank). The CDB has overtaken the World Bank has the single biggest provider of international development finance. However, there are concerns over the environmental and social impacts of China’s development assistance, which may lack the stringent loan conditions from western institutions. This is particularly acute in energy projects, with coal projects backed by China having an estimated annual social cost of US$ 27 billion. (*Financial Times*, 17 May 2016)

➢ The East African Community (EAC), comprising 6 countries, is Africa’s most integrated trading bloc. It agreed a customs union in 2005, and a common market in 2010. And, according to an analysis from the International Growth Centre, the region is richer and more peaceful as a result. The analysis found that bilateral trade was 213% higher in 2011 that it would otherwise have been, despite the ongoing 51 non–tariff barriers. The authors argue that full implementation would double income gains, but are sceptical of the EAC’s aim of creating a common currency by 2024, arguing that it would have little benefit on trade. The IMF found that unless the EAC’s economies converge, a single currency would mean that wage adjustments would be the only way to absorb economic shocks – and Greece can attest to the resultant economic hardship. However, the authors conclude that interdependence reduces the risk of war, and in a continent where national economies are small, regional trade blocs provide more economic clout. (*The Economist*, 11 June 2016)

➢ Following the UK’s vote in June to leave the European Union (“Brexit”), the value of the British pound (GBP) fell sharply. Among the major repercussions from this fall is a drop in the value of UK overseas aid, which fell by US$ 1.4 billion in one night. To place this in context, US$ 1.4 billion is almost equivalent to the National Security Council–led Prosperity Fund’s budget of US$ 1.3 billion, which is ear–marked to place 11 million children in school, bring clean water and sanitation to 60 million people, save 1.4 million lives through immunisation, and improve nutrition for 50 million people. Moreover, the UK pledges to spend a fixed proportion of its GDP on development assistance, so if economic growth falters or the UK experiences a recession then the value of UK overseas aid will also decrease. The EU budget for development assistance will also be affected, as the UK channels 2 billion Euros (US$ 2.2 billion) into its budget for overseas aid, and every US$ 1 that the UK spends on EU development assistance is matched by US$ 6 from other EU member states. (*Humanosphere*, 24 June 2016)

➢ Despite recent events in Turkey, globally coups have become increasingly rare. This is highlighted by recent research from the University of Kentucky, which shows that attempted coups peaked in the mid–1960s, with two peaks in the mid–1970s and early 1990s (the end of the Cold War). However, this analysis also shows that coups are more likely to succeed, with a record 60% now succeeding, compared to 20% at the start of the 21st century. Separate research from the Stockholm School of Economics suggests that the economic consequences of coups are particularly dire when democracies are overthrown. These typically lower economic growth by 1–1.3% a year over the following decade. This runs counter to the argument that Chile and Turkey’s economic growth is linked to their coups in 1973 and 1980 which brought in economic liberalisation; the research found that post–coup economic growth was lower than pre–coup economic growth, and that growth rates were lower–than–expected. Coups tend to cause increased indebtedness, deteriorating external financial positions, a higher chance of economic crises, and reduced social spending shifts priorities from the wider population to the country’s elite, with knock–on effects on health and education. However, coups which successfully overthrow autocratic leaders are often economically beneficial. The economic impact of Turkey’s failed coup depends on whether Turkey is considered a democracy; its president Recep Tayyip Erdogan was democratically elected, but subsequently centralised power and made Turkey’s intelligence services accountable to him alone. Turkey’s ranking for freedom of expression and beliefs is now more akin to an autocracy, while its press freedom is the lowest of any democracy and is in line with a mid–ranking autocracy. (*Financial Times*, 20 July 2016)

➢ China is the world’s third–largest economy, accounting for 15% of global output, but its contribution to global economy growth could be yet more significant. It is estimated that China contributes 25–30% to total global economic growth. However, much of this growth will fuel China’s own economic growth, raising its citizens’ living standards. Globally the benefits will be felt at the margin, as more and higher–value exports from China ensures that world–wide people can access more and better products, while Chinese consumption of imported goods will support employment in other countries. India’s economy is growing faster than China’s, but this will have less global impact because it is growing from a lower base, and the extra size of its economy each year is very much smaller than China’s economy. (*Forbes*, 30 October 2016)

## Energy

➢ The International Energy Authority (IEA) has stated that Middle Eastern oil producers, such as Saudi Arabia and Iraq, have the largest share of world oil markets since the Arab fuel embargoes of the 1970s. The collapse in oil–prices has reduced output from higher–cost producers (eg, Canada, Brazil), leading to a surge in demand for Middle Eastern crude oil. Unlike the 1970s oil crisis, OPEC producers – led by Saudi Arabia and its Gulf allies – decided to maintain their output to defend market share, rather than reducing output to increase prices. This has led to surging demand, and thwarted efforts to reduce greenhouse gas emissions as more consumers buy fuel–guzzling cars. In the USA, more than 2.5 times as many sport utility vehicles (SUVs) are now bought compared to standard cars; this is echoed in China, which is rapidly adopting the US’s taste for fuel–heavy cars, with more than 4 times as many SUVs being purchased. China is now the focus in the global growth in oil demand, and in 2015 overtook the USA as the world’s biggest oil importer. Mr Fatih Birol, IEA’s executive director, has called for stricter fuel efficiency targets to reduce demand, and argues that the rise of shale oil will not reduce the Middle East’s dominance of global oil markets. (*Financial Times*, 7 July 2016)

➢ On the face of it, energy efficiency is a highly appealing policy, which appears to reduce costs, create jobs and save the planet. However, the UK recently ended its US$ 316 million energy efficiency loan project, after its National Audit Office concluded that people were not signing up, nor delivering cost–effective energy efficiency measures for those who did. On a similar note, there is no evidence that houses built under California’s new building energy codes use less energy than earlier houses. This is partly because of the “rebound” effect, whereby improving energy efficiency can lead to higher energy consumption. According to researchers at the Copenhagen Consensus Center, it would cost US$ 3.2 trillion to achieve the UN target of doubling the rate of improvement in energy efficiency – the International Energy Agency expects expenditure to be US$ 550 billion by 2035. An alternative approach to tackling climate change is to develop green technology to the point where it is cheaper than oil, gas or coal. This would be achieved not via subsidies, but by increasing research and development expenditure to make the next generations of wind, solar and biomass energy cheaper and more effective. If 0.2% of global GDP was devoted to green–energy R&D, then the chances of breakthrough are significantly increased. This would have benefits 11 times greater than the amount spent – compared to 2.4–3 times under the current method. (*Project Syndicate*, 20 July 2016)

➢ Argentina’s Supreme Court has ruled that the government must hold public hearings before cutting energy subsidies and increasing domestic utility rates. Since his election in November 2015, President Mauricio Macri has cut gas subsidies, which caused a sharp upturn in domestic heating bills – up to 400% – following an unusually cold winter, and the cost of electricity has increased by up to 700%. The court ruled that Argentina’s government had violated the law on natural gas which requires that hearings are needed to secure the constitutional right to information, consultation and participation for users and consumers. This will restore residential gas prices to their original levels, and the Macri government must now hold public comment sessions before it can implement further increases on residential gas rates. (*TeleSUR*, 18 August 2016)

➢ According to the UK government, Électricité de France (EDF) SA’s planned Hinkley Point C nuclear plant will cost British consumers US$27 billion in subsidies over the lifetime of the French company’s contract with the UK. EDF plans to build two nuclear reactors at Hinkley Point, which will generate 7% of the UK’s electricity, with China’s General Nuclear Power Corporation providing 33% of funding. This project has proved controversial, in part due to concerns over China’s involvement, and that the contracted price is more than twice the current wholesale rate. It will add an estimated US$ 20 to each household’s annual electricity costs. However, the government noted that nuclear power stations are an important part of ensuring the UK’s low–carbon energy security, large–scale solar and on–shore wind could not produce the same amount of electricity as Hinkley Point without significant upgrades to the national grid, and relying on gas would endanger the UK’s carbon emission targets. (*Business Insider*, 29 September 2016)

➢ Saudi Arabia is the world’s second–largest producer of oil, which is a major contributor to climate change, but is now turning its attention to green energy generation. This may be prompted by the current oversupply of oil and dwindling demand due to the global economic slow–down and the growth of renewable energy. Khaled al–Faleh, the kingdom’s oil minister, confirmed that Saudi Arabia is making major investments in renewables, with the goal of producing 9.5 MW from renewable energy, mainly solar and wind sources. However, these investments are aligned to further investments in hydrocarbons and petrochemicals. The Saudi oil giant, Aramco, confirmed that it intends to play a key role in country’s drive to become a top clean–energy producer. Other oil giants, including Aramco, are moving to establish investment funds to develop carbon mitigation technologies in addition to renewable energy. A public announcement of this plan is expected to coincide with the formal launch of the 2015 Paris Agreement to phase out man–made greenhouse gases during this century. (*New Arab*, 2 November 2016)

## Environment

➢ Faced with desiccated pastures in Ethiopia’s Somali region in November 2015, many pastoralists traveled to Somaliland (an internationally unrecognised but *de facto* sovereign nation separate from Somalia) – a journey of hundreds of kilometres – in the hope of finding rain and fresh pasture. However, they found insufficient rain and pastures to support the number of arrivals, and thousands of cattle, sheep, goats and camels perished. This drought, named *mulia (*ie, “that which erases everything on the ground”) is the most severe for 50 years, although there is no repeat of the 1984 famine, which caused the deaths of more than 1 million people. To date, Ethiopia’s effective emergency response has staved off disaster, but faced with climate change and increasingly frequent, severe droughts in the Horn of Africa, lifestyle changes may be unavoidable and the traditional way of life of Ethiopia’s pastoralists may be increasingly unsustainable. “In areas affected by climate change, you just can’t have the same numbers of people surviving in these conditions – it’s beyond the control of the people or the government,” says John Graham, Ethiopia country director for Save the Children. This is recognized by the pastoralists themselves, who believe that a government re–stocking program, or a government re–settlement program to more reliable areas may give them a chance to preserve their way–of–life. (*IRIN*, 3 June 2016)

➢ Carbon capture and storage can reduce CO_2_ emissions and is part of the battle against climate change, but there are concerns over storing it safely and preventing leaks. The small–scale CarbFix project in Iceland pumped a mixture of CO_2_ and water into underground basalt rocks. This caused the acidic mixture to dissolve the rocks’ calcium magnesium and turn into limestone, and within two years 95% of CO_2_ was captured and successfully converted to limestone – an acceleration of a natural process. The main drawback to this new technology is that it is currently twice as expensive as injecting CO_2_ into old wells, although the ocean floor is a promising source of basalt for storage. Ken Caldeira, a climate scientist at the Carnegie Institution for Science, described the possibility of low–cost scale–up of the technology as “very good news”. (*Japan Times*, 10 June 2016)

➢ Iceland’s capital city, Rejkavik, is aiming to become carbon–neutral by 2040. The city already produces all its electricity through hydroelectric power and houses are heated geothermally, so its greenhouse gas emissions are low in comparison with other cities. This means that other sectors, primarily transport, must be at the forefront of its drive to become carbon–neutral. The city has set out plans to increase public transport usage from 4% to 12%, increase the ratio of pedestrians and cyclists from 19% to over 30% by 2030, increase the use of bicycles and buses as primary transport methods, and increase the availability of charging stations for electric cars. “Achieving carbon neutrality is entirely within the realm of what is possible for cities. It sets an ambitious target, while also recognising that certain steps towards climate neutrality may be easier to attain than others,” said Monika Zimmerman, the Deputy Secretary of Local Government for Sustainability. (*Cities Today*, 21 September 2016)

➢ The USA’s Environmental Protection Agency (EPA) has been rebuked over its failure to act swiftly in warning the residents of Flint Michigan that their water was contaminated with lead. Flint’s water supply became contaminated after its water source was switched to the Flint River in 2014, partly to save money. Arthur A Elkins, the Inspector–General for the EPA, noted that EPA officials had enough information and authority to issue an emergency order in June 2015, when officials knew that systems designed to protect drinking water from lead contamination were not in place, residents had reported abnormalities in the water, and some test results showed lead levels above the federal action level. However, the EPA did not issue an emergency order until January 2016, causing these conditions to persist. However, Gina McCarthy of the EPA told a Congressional committee in March 2016 that the agency had been repeatedly misled by state employees, and was not responsible for creating the initial lead problem, noting that state officials had agreed to add corrosion controls to Flint’s water but delayed in doing so. (*New York Times*, 20 Oct 2016)

➢ Middle–class residents are increasingly abandoning New Delhi (India’s third–largest city), as particulate levels reached 30 times the safe limit recommended by the World Health Organization, and Anil Madhav Dave, India’s environment minister, warned of an “emergency situation”. Arvind Kerjriwal, the city’s chief minister, announced some measures to combat pollution, including the temporary closure of construction sites, banning diesel generators for 10 days except for hospitals and emergencies, closing a coal–fired power station etc. An earlier scheme to restrict car usage reduced pollution levels, but its effects were limited by cars only contributing 25% of small particle emissions – another 25% is due to the illegal burning of crops by farmers in other states, which the government has failed to tackle. According to the World Bank, the annual cost of India’s air pollution is more than US$ 40 billion – or 3% of its GDP – and economists warn that outward migration caused by pollution could cause serious economic damage to New Delhi’s economy. However, one area of the economy is booming – the sales of air purifiers. (*Financial Times*, 6 November 2016)

## Food, Water and Sanitation

➢ Recent innovations in farming –so–called “smart farming” – has led to farming becoming more like factories, as tightly–controlled operations which product reliable products, increasingly independently from the natural world. Improved understanding of DNA makes genetic manipulation of plants and farm animals possible, which may be more palatable to consumers than the shifting of whole genes between species. These new technologies are likely to improve profits by cutting costs and increasing yields, and lower prices will benefit consumers. They may also help answer the challenge of feeding the world’s population as it grows from 7.3 billion to 9.7 billion by 2050. According to the UN’s Food and Agriculture Organisation, this would require a 70% increase in production – which can realistically only be met by higher yields, as most land suitable for farming is already under cultivation. Technologies such as farm–management software, hydroponics and drones increase yields, fish–farming may be brought inland thanks to artificial ecosystems, while cultivated land can be increased by using old buildings (“vertical farms”) and underground tunnels as farmland. Researchers have also produced laboratory–grown muscle cells which can be developed into meat products, and synthetic egg whites, raising the possibility of increasing meat and egg consumption without increasing livestock production. Rice yields in China could increase if the precision agricultural techniques used in North America’s arable farms could be adapted. Technology could also increase Africa’s agricultural yields, illustrated by the NextGen Cassava Project, which increases the crop’s yields and nutritional value while reducing their susceptibility to disease. (*Economist*, 9 June 2016)

➢ In recent years, drought and water shortages have affected animal husbandry in Vietnam’s central regional, reducing livestock’s natural food sources and causing many deaths among cattle. The government’s Animal Husbandry department will assess the future of animal breeding during prolonged heatwaves and drought, and include new zones for animal husbandry. The breeding of sheep, ostriches, goats, honeybees and silkworms have helped increase farmers’ incomes, and offset crop losses caused by drought and saltwater intrusion. In Khánh Hoà province, officials are working on a plan for agricultural restructuring, with a focus on animal husbandry, especially on breeding animals that can survive drought conditions. (*Việt Nam News*, 11 June 2016)

➢ In March, China discontinued its minimum price policy for grain, whereby the government bought grain at above–market rates, thus distorting market prices and leading to expensive grain being hoarded in silos. The end of this policy held down prices and lessened crop acreage, in the expectation that grain would be released from storage. However, grain imports and prices are increasing, causing suspicions that much of the stored grain is unsuitable for use. Demand for corn from China’s livestock industry has driven record corn imports in April and May 2016. China and Russia have also pledged to invest US$ 185 million in grain elevators and a terminal in Russia to support China’s future grain purchase – another indicator of China’s long–term concerns over its grain supplies. (*Financial Times*, 11 July 2016)

➢ A global study of heights over the past century has found that the tallest nations (including Estonia, Denmark, Serbia and the Czech Republic) are all found in Europe. The Netherlands has the highest average height for men (182.5 cm), and Latvia has the highest average height for women (170 cm). The study also found that English–speaking countries – primarily the USA – are falling behind other high–income countries in Europe and Asia Pacific. The relative decline, coupled with these nations’ poor record in obesity, highlights the need for good life–long nutrition. However, the average heights for young men and women have decreased by up to 5 cm in some countries in sub–Saharan Africa, including Sierra Leone, Uganda and Rwanda. “The average height of some nations may even be shrinking while others continue to grow taller … This confirms we urgently need to address children and adolescents’ environment and nutrition on a global scale,” says Majid Ezzati, a global health researcher at Imperial College London. (*New Scientist*, 26 July 2016)

➢ India’s Urban Development Minister, Venkaih Naidu, believes that sanitation will be an election issue, as urban residents are more likely to support candidates with strong policies on hygiene. He is also confident that India’s National Democratic Alliance (NDA) government will achieve its “Clean India” mission, including making India open–defecation–free (ODF) by 2019. Prime Minister Modi has issued calls to make the Clean India campaign a national movement, and Mr Naidu stressed that behavior changes toward using toilets and putting waste into litter bins is key to its success. He noted that the campaign is on–track to meet its target on constructing household toilets, and Gurjarat and Andhra have already declared themselves ODF in urban areas, and Kerala is set to make a similar announcement. To date, 405 cities have been declared as ODF, and nearly the same amount are expected to be ODF by March 2017. However, he is concerned about the bigger challenge of dealing with India’s refuse, but noted that the government plans to tackle it by turned 65 million tonnes of urban refuges into compost and electricity. (*First Post*, 29 October 2016)

## Peace and Human Rights

➢ Today, the USA consumes six times more prescription opioids per person than it did 20 years ago, often for chronic, non–terminal pain. Over–prescription, haphazard clinical practice and spotty oversight have led to addiction and deaths. However, other countries face the opposite problem. *The Lancet* estimates that 40 Russians have committed suicide in a single year because of unbearable pain, and globally opioid shortages are more common than the overuse seen in some countries. Nigeria began importing morphine in 2012, which has improved access, but it is only available from Lagos and smaller hospitals outside the capital struggle to afford the journey to buy it. Palliative care is difficult to access in most developing countries, and facilities are concentrated in cities, making it much harder for rural patients to receive treatment to alleviate their suffering. This situation is mirrored in Colombia, which produces its own opioid painkillers, but some regional governments cannot afford to buy them, or view them as a low priority. Moreover, the campaigning group Human Rights Watch has highlighted the paucity of training in palliative care, meaning that few doctors know how to prescribe pain–killers safely, and the bureaucracy faced by cancer patients to obtain pain relief in some countries. Opioid drugs are relatively cheap to make, but tariffs, import licenses and high costs for small–scale production means that morphine is twice as expensive in developing countries, while low profit margins on opioids give little incentive to drug firms to bring them to new markets. And often, pain management is simply not a priority in the developing world, where many governments focus on life–threatening epidemics. (*Economist*, 28 May 2016)

➢ Uganda has been a safe haven for many South Sudanese people during the country’s 60 years of turmoil. However, the Adjumani district in northern Uganda has seen new arrivals increasing to 250/day from an earlier average of 160/day. There are more than 132 000 refugees in the area – the highest concentration in the country – and overall Uganda is home to 510 000 refugees. Uganda’s refugee model avoids traditional camps far from communities, but provides refugees with a basic ration, plots of land and materials with which to build their new lives. This has been celebrated as progressive, forward–looking, building self–sufficiency and integrating refugees into their host community. However, this is being stretched to the limits, as increasing number of refugees mean that land plots are shrinking which increases their reliance on aid – the very thing it was supposed to avoid. Ethnic tensions are also increasing as other communities have been caught up in South Sudan’s struggle, and refugees’ ability to trade their goods in larger markets outside the settlements is compromised by security concerns, which would lower their incomes. As South Sudan’s prospects for peace look increasingly grim, Uganda’s model of refugee integration faces further strains. (*IRIN*, 13 June 2016)

➢ Syrian teenager Yusra Mardini will become the first member of the Refugee Team to compete in the Rio 2016 Olympic Games, in the women’s 100–metre butterfly heats. Speaking in Rio alongside the other athletes from the Refugee Team, Yusra said “a lot of things have happened in our lives, but remember that life will not stop for you.” The 10–strong Refugee Team comprises judoka [judo contestants], swimmers and track–and–field athletes. Yusra said that her goal is for everyone to understand that most refugees are normal people who have had to flee their homelands, and that each member of the Refugee Team is very aware of the opportunity to present a positive, inspiring picture of refugees. “They have dreams in their lives, and had to go. Everything is about trying to get a new, better life and by entering the stadium, we are encouraging everybody to follow their dreams,” she said. (*Huffington Post*, 8 August 2016)

➢ Civilians cannot access medical care in the besieged Afghan city of Lashkar Gah – the capital of Helmand province – and agencies are preparing for a possible Taliban takeover. Recent fighting has displaced 10 000 people, and despite government assurances that the city will not fall to the Taliban, aid agencies are making plans for this eventuality. Médecins Sans Frontières (MSF) has “a massive casualty plan prepared and ready to implement immediately in the case of a further deterioration of the security situation”. MSF is in contact with militants, plus government and allied forces, and shares its GPS co–ordinates with all sides to help protect patients and staff. When the Taliban briefly took over Kunduz in 2015, it allowed MSF to treat war–wounded people, until its trauma center was destroyed by a US airstrike. In early August, MSF treated 25 war–wounded people in the province, but noted that fighting makes it extremely difficult to travel to Lashkar Gah for treatment. A 15–year old girl died from meningitis, as road blocks and check–points cause delays in reaching hospital, and other people are also likely to be suffering with, and even dying from, treatable conditions because they can’t reach hospital. Aid agencies are distributing food, water and supplies to displaced people in Lashkar Gah, but if the city falls to the Taliban civilians may be trapped, as roads are inaccessible and threaten the delivery of aid. (*IRIN*, 17 August 2016)

➢ The European Union confirmed that it will not be introducing a UN resolution condemning Myanmar’s human rights record – the first time in 15 years – and praised the country’s progress under the leadership of Aung San Suu Kyi. The EU acknowledged the tentative steps toward addressing violence between the majority Buddhists and Muslim Rohingyas in the state of Rakhine. The Rohingyas are often perceived as illegal immigrants from Bangladesh, even though many have lived in Myanmar for decades, most were disenfranchised in the 2015 election, and around 125 000 are confined in temporary camps. The US ambassador to the UN, Samantha Power, lauded Suu Kyi’s commitment to stand firm against intolerance and her pledge to grant citizenship to all those entitled to it as “powerful and important”. In Suu Kyi’s first address to the UN General Assembly, she defended her government’s efforts to resolve the Rohingya crisis, and stated that the government would persevere in its efforts to bring peace to Rakhine, and will stand firm “against the forces of prejudice and intolerance.” (*Reuters*, 24 September 2016)

## Science and Technology

➢ The recent Ebola outbreak in West Africa shows the importance of fast and accurate diagnostics, especially in countries with weak health systems. Redemption Hospital in Monrovia, Liberia used one of the first machines – GeneXpert – for Ebola testing, which gave cheap and accurate results within 90 minutes, and was crucial in reducing false Ebola scares and restoring confidence among Monrovian residents. Other portable “point of care” testing kits can be used to screen for other infectious diseases such as tuberculosis, HIV and malaria, and can help measure the effectiveness of health campaigns. Tests can be administered on–site, but diagnoses sometimes take place thousands of miles away, for example local doctors in 19 African health centers are connected online with 100 volunteer specialists in Europe. Portable, user–friendly, low–energy diagnostic kits are especially important in areas with limited access to power or laboratory equipment. The next step for diagnostic technology is to test for multiple causes of a symptom, such as a fever. (*BBC*, 20 September 2016)

➢ Vaccine production often involves cultivating vaccine–producing bugs in secure, centralised areas, followed by global distribution. Security is needed to prevent organisms, particularly modified organisms, escaping into the wider environment. This approach works well when they are sent to places that have proper infrastructure to handle vaccines, such as reliable refrigerating equipment to keep them cool (the “cold chain”), but often in areas where they are most needed these facilities are less available. However, if vaccines could be made on site, it would simplify and shorten the distribution chain. James Collins from the Massachusetts Institute of Technology may have resolved this, by devising a vaccine factory that consists of the cellular components needed to synthesize the required molecules, rather than whole cells, in a way that can be freeze–dried for easy transport and storage – with no risk of escaped bugs. Mr Collins and colleagues looked at how solutions containing protein–production machinery responded when given DNA templates that encoded the antigens used to make various vaccines, and found that all were readily activated by the rehydrated cellular machinery. If this approach can be commercialised, it could greatly simplify vaccine manufacturing and distribution. (*Economist*, 24 September 2016)

➢ The 2016 Nobel Prize in physiology or medicine was awarded to Japanese biologist Yoshinori Ohsumi, for his work on uncovering how the body’s cells deal with and recycle waste, known as autophagy. Cells use this process to degrade some of their contents and clear them away or recycle them, and Dr Ohsumi identified the first genes required for autophagy in yeast and subsequently contributed to the understanding of this works in humans and animals. This knowledge may help develop treatments for conditions such as Alzheimer Disease, Parkinson Disease and cancer. Dr Ohsumi said his research was important because it highlights how cells cannot function without “quality control,” and shedding and recycling substances it no longer needs. “We create proteins and destroy them, and again create and destroy, and that’s what makes life exist,” he says. (*Wall Street Journal*, 3 October 2016)

➢ More than 1% of US children are diagnosed with an autistic spectrum disorder, and no treatment has been shown to effectively address the core symptoms of autism. However, the Preschool Autism Communications Trial (PACT) enrolled 152 children aged 2–4 years with autism – many with severe symptoms – to test the effectiveness of early interventions, which are believed to have a greater impact as symptoms are less severe and the brain is at an earlier stage of development. PACT taught the parents of these children how to interact more effectively with their children. The results of the trial, published in *The Lancet,* shows that six years after the year–long course the children still showed improved social communication and reduced repetitive behaviors, and fewer were considered to have severe autism in comparison with a control group. John Constantino, a child psychiatrist at Washington University describes this as “monumentally important” as there was previously little evidence that early–stage interventions are beneficial. Despite improving communications skills and decreasing repetitive behavior, the therapy did not lessen children’s anxiety – a key symptom of autism – and underscores the importance of developing higher–impact interventions. (*Nature News*, 25 October 2016)

➢ The Ebola virus appears to have mutated in 2014, which made it less likely to infect its traditional host of bats, and more likely to infect humans. The mutation, known as A82V, spread quickly beyond Guinea, where Ebola first broke out, and was involved in the vast majority of cases. However, the research teams who identified the mutation emphasize that it was not directly responsible for Ebola’s spread, which is more closely linked to the movement of infected people in urban areas, and the lack of proper burials. It is also unclear if A82V is more fatal; certainly people infected with the mutant strain were 27% more likely to die, but this may be due to limited access to health care, as the outbreak took hold when infected people were moving to urban areas. Even without the A82V mutation, some strains of Ebola have mutations that make it easier for them to spread from bats to humans. Although Ebola is currently halted, and A28V has disappeared, the research highlights that the next outbreak could include different mutations that make it easier for the virus to spread – constant vigilance, monitoring and rapid response is needed. The A82V mutation could have been prevented if the Ebola outbreak had been stopped earlier. (*The Atlantic*, 3 November 2016)

